# Clinical characteristics of persistent or recurrent pneumonia combined with airway malacia in children

**DOI:** 10.1111/crj.13767

**Published:** 2024-04-29

**Authors:** Zhuxia Li, Xinxing Zhang, Huiquan Sun, Chuangli Hao, Xuejun Shao, Jun Xu, Xin Zhang, Yuqing Wang

**Affiliations:** ^1^ Department of Respiratory Medicine Children's Hospital of Soochow University Suzhou China; ^2^ Department of Laboratory Medicine, Department of Clinical Laboratory Children's Hospital of Soochow University Suzhou China

**Keywords:** airway malacia, children, clinical characteristics, persistent or recurrent pneumonia

## Abstract

**Objective:**

The aim of this study is to investigate the clinical characteristics and pathogens involved in persistent or recurrent pneumonia combined with airway malacia in children.

**Methods:**

We retrospectively reviewed the information of children hospitalised with persistent or recurrent pneumonia, including clinical presentations, laboratory examination results and pathogens.

**Results:**

A total of 554 patients were admitted, 285 (51.44%) of whom were found to have airway malacia. There were 78 (27.37%), 166 (58.25%) and 41 (14.39%) patients with mild, moderate and severe malacia, respectively. Patients with airway malacia were younger than those without malacia (6.0 vs. 12.0 months, *p* < 0.01) and were more likely to present with wheezing (75.07%), fever (34.39%), dyspnoea (28.77%), cyanosis (13.68%) and wheezing in the lungs (78.95%). The incidence of preterm delivery, oxygen therapy, paediatric intensive care unit (PICU) admission and mechanical ventilation was higher, and the hospital stay (11.0 vs. 10.0 days, *p* = 0.04) was longer in these patients than in those without malacia. Patients with severe airway malacia were more likely to undergo oxygen therapy, PICU admission, mechanical ventilation and have multiple malacia than were those with mild or moderate malacia. *Mycoplasma pneumoniae* (30.18%) was the most common pathogen.

**Conclusion:**

Severe airway malacia likely aggravates conditions combined with pneumonia. The proportion of multisite malacia was greater in severe airway malacia patients.

## Graphical abstract

Airway malacia is one of the underlying causes of recurrent or persistent pneumonia. Patients with airway malacia were more likely to receive oxygen therapy, mechanical ventilation and paediatric intensive care unit (PICU) admission than were those without malacia. Airway malacia probably aggravates conditions combined with pneumonia.

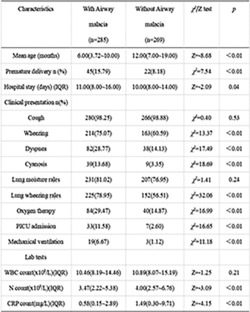

## 1 INTRODUCTION

Community‐acquired pneumonia (CAP) is a major cause of morbidity and mortality in childhood.^1^ Some of these children suffer from recurrent or persistent pneumonia. Currently, the prevalence of recurrent or persistent pneumonia in children is still unknown, although a previous study reported that 7.7%–9% of children with CAP had recurrent pneumonia.^2^ Recurrent or persistent pneumonia is a common clinical presentation and poses a significant challenge for paediatricians.

There are various underlying causes for recurrent or persistent pneumonia, including neurological disorders, congenital heart disease, allergies and gastroesophageal reflux.^3^, ^4^ Heffelfinger et al.^5^ found that children with recurrent pneumonia presented with other diseases (such as airway malacia and foreign body aspiration) and whether their coexistence in a child represents a causal association is not always clear. A clinical study of children who underwent bronchoscopy due to recurrent lower respiratory tract infection revealed that 52% of the children had airway malacia.^6^ Gokdemir et al.^7^ reported that malacia disorders were the most common cause of recurrent or persistent pneumonia in children. Malacia is often described as either primary malacia due to an inherent abnormality of the tracheal cartilage and trachea or secondary malacia due to structural abnormalities of the mediastinum (such as tracheoesophageal fistula and aortic arch malformations).^8^, ^9^, ^10^ A retrospective review of 219 children diagnosed with tracheobronchomalacia, including 161 with primary malacia and 58 with secondary malacia, revealed that recurrent respiratory tract infections were one of the main manifestations.^8^ Based on a previous study, we hypothesised that airway malacia was probably related to recurrent or persistent pneumonia and that there were differences in pathogens between children suffering from recurrent or persistent pneumonia with and without airway malacia. The aim of this study was to investigate the relationship between airway malacia and recurrent or persistent pneumonia.

## 2 METHODS

### 2.1 Patients

Children who were under the age of 5 years old that were hospitalised with persistent or recurrent pneumonia between June 2016 and December 2020 and who underwent bronchoscopy were enrolled. Persistent pneumonia is defined as the persistence of symptoms and radiographic abnormalities in a child for more than a month despite appropriate therapy^7^; recurrent pneumonia is defined as at least two pneumonia episodes within 1 year.^2^ Patients with bronchopulmonary dysplasia, a history of mechanical ventilation at birth, congenital heart disease, inherited metabolic disorders, immunodeficiency disease, neuromuscular disease, foreign body aspiration or incomplete clinical data were excluded.

### 2.2 Bronchoscopy and bronchoalveolar lavage fluid (BALF) collection

Flexible bronchoscopy was performed after providing written parental consent. The entire procedure was carried out under local anaesthesia. Atropine (0.01–0.02 mg/kg) and midazolam (0.1–0.3 mg/kg, maximum 4 mg/dose) were administered intramuscularly 30 min before the procedure. Lidocaine (2%) was administered three to four times via nasal and transoral sprays, and 1% ephedrine and furacilin were dropped into the right nose. The bronchoscope was inserted through the right nostril, and local anaesthesia was administered on the endotracheal mucosal surface. Throughout the procedure, respiratory rate, heart rate and oxygen saturation were monitored.

The bronchoalveolar lavage was performed three times at 37°C in normal saline (1 mL/kg per lavage for weight <20 kg, 20 mL per lavage for weight ≥20 kg). BALF obtained by suction at a negative pressure of 13.3–20.0 kP (100–150 mmHg) was stored in a sterile sputum collector and sent for examination.

According to the extent of the malacia, the severity of the malacia was classified as mild (one‐third to one‐half collapse), moderate (one‐half to three‐quarters collapse) or severe (more than three‐quarters collapse).^11^

### 2.3 Detection of pathogens

Direct immunofluorescence was used to detect respiratory syncytial virus (RSV), influenza virus A, influenza virus B (IV A, IV B), parainfluenza (PIV) I, PIV II, PIV III and adenovirus (ADV). Reverse transcriptase polymerase chain reaction (RT‐PCR) was used to detect human metapneumovirus (hMPV), human bocavirus (hBoV) and human rhinovirus (hRV) genes. *Mycoplasma pneumoniae* (MP) DNA was detected by Fq‐PCR and PCR amplification.

BALF was inoculated onto selected media and incubated for 18–24 h. Bacteria were identified using quantitative analysis. The cut‐off point for a positive quantitative culture of pathogenic bacteria was more than 10^3^ colony‐forming units/mL.

### 2.4 Data collection

Demographics (age, sex), clinical features (symptoms, signs, hospital stay, mechanical ventilation, oxygen therapy and paediatric intensive care unit [PICU] admission) and laboratory test results (white blood cell [WBC] count, neutrophil count and C‐reactive protein [CRP]) were collected from the enrolled children.

### 2.5 Statistical analysis

SPSS 25.0 was used for data analysis. Nonnormally distributed data are expressed as medians (interquartile ranges). The Mann–Whitney *U*‐test and Kruskal–Wallis *H*‐test were used for comparisons of quantitative variables among different groups. The chi‐square test was used for categorical data. *P* < 0.05 was considered to indicate statistical significance.

## 3 RESULTS

### 3.1 Patient characteristics

A total of 554 patients with persistent or recurrent pneumonia were included, of which 446 had persistent pneumonia and 108 had recurrent pneumonia. Airway malacia was detected in 285 children (51.44%), and no airway malacia was observed in 269 (48.56%). Additionally, 554 patients had no other airway malformations. In the airway malacia group, there were 202 males and 83 females; 242 patients (84.91%) were aged under 1 year, 36 patients (12.63%) were aged between 1 and 2 years, 6 patients (2.11%) were aged between 2 and 3 years and 1 patient (0.35%) was >3 years. There were 189 males and 80 females without airway malacia.

### 3.2 Bronchoscopy observation

There were 53 (18.60%), 88 (30.88%) and 144 (50.52%) patients with tracheomalacia, tracheobronchomalacia and bronchomalacia, respectively. Of the patients with bronchomalacia, 44 patients had left‐sided bronchomalacia, 59 patients had right‐sided bronchomalacia and 41 patients had bilateral bronchomalacia. According to the malacia scale, 135 patients (47.37%) had focal malacia, and 150 patients (52.63%) had multiple malacia. With regard to the extent of malacia, 78 patients (27.4%) had mild malacia, 166 patients (58.2%) had moderate malacia and 41 patients (14.4%) had severe malacia.

### 3.3 Clinical characteristics of children with pneumonia with or without airway malacia

Children with airway malacia were younger than those without airway malacia (6.0 vs. 12.0 months, *p* < 0.01). Children with airway malacia were more likely to present with wheezing (75.07%), fever (34.39%), dyspnoea (28.77%) and cyanosis (13.68%, all *p* < 0.05). The incidence of preterm delivery (15.79%), oxygen therapy (29.47%), PICU admission (11.58%) and mechanical ventilation (6.67%) was higher in children with airway malacia than in those without airway malacia, and the hospital stay was longer (11.00 vs. 10.00 days) (all *p* < 0.05; Table 1).

**TABLE 1:** Clinical characteristics of pneumonia children with and without airway malacia.

Characteristics	With airway malacia (*n* = 285)	Without airway malacia (*n* = 269)	*χ* ^2^/Z test	*p*
Mean age (months)	6.00 (3.72–10.00)	12.00 (7.00–19.00)	Z = −8.68	<0.01
Premature delivery *n* (%)	45 (15.79)	22 (8.18)	*χ* ^2^ = 7.54	<0.01
Hospital stay (days) (IQR)	11.00 (8.00 ~ 16.00)	10.00 (8.00–14.00)	Z = −2.09	0.04
Clinical presentation *n* (%)				
Cough	280 (98.25)	266 (98.89)	*χ* ^*2*^ = 0.40	0.53
Wheezing	214 (75.09)	163 (60.59)	*χ* ^*2*^ = 13.37	<0.01
Dyspnea	82 (28.77)	38 (14.13)	*χ* ^*2*^ = 17.49	<0.01
Cyanosis	39 (13.68)	9 (3.35)	*χ* ^*2*^ = 18.69	<0.01
Lung moisture rales	231 (81.05)	207 (76.95)	*χ* ^*2*^ = 1.41	0.24
Lung wheezing rales	225 (78.95)	152 (56.51)	*χ* ^*2*^ = 32.06	<0.01
Oxygen therapy	84 (29.47)	40 (14.87)	*χ* ^*2*^ = 16.99	<0.01
PICU admission	33 (11.58)	7 (2.60)	*χ* ^*2*^ = 16.65	<0.01
Mechanical ventilation	19 (6.67)	3 (1.12)	*χ* ^*2*^ = 11.18	<0.01
Lab tests				
WBC count (×10 ^9^ /L) (IQR)	10.46 (8.19–14.46)	10.89 (8.07–15.19)	*Z* = −1.25	0.21
*N* count (×10 ^9^ /L) (IQR)	3.47 (2.22–5.38)	4.00 (2.57–6.76)	*Z* = −3.09	<0.01
CRP count (mg/L) (IQR)	0.58 (0.15–2.89)	1.49 (0.30–9.71)	*Z* = −4.15	<0.01

Abbreviations: CRP, C‐reactive protein; IQR, inter‐quartile range; PICU, paediatric intensive care unit; WBC, white blood cell.

### 3.4 Comparison of the clinical characteristics of pneumonia in children with different extents of airway malacia

To evaluate the differences among different extents of airway malacia, we divided the children into three different groups (Table 2). In the severe airway malacia group, the proportions of premature infants (29.27%) and hospital stays (15.00 days) were greater than those in the mild malacia group (all *p* < 0.05). Children with severe airway malacia were more likely to undergo oxygen therapy (63.41%), PICU admission (24.39%), mechanical ventilation (21.95%), and have dyspnoea (63.41%) and multiple malacia (85.37%) than those with mild or moderate malacia (all *p* < 0.05).

**TABLE 2:** Clinical characteristics of children with different extent of airway malacia.

Characteristics	Mild (*n* = 78)	Moderate (*n* = 166)	Severe (*n* = 41)	*χ* ^2^/*H*‐test	*p*
Mean age (months)^a^	6.77 (4.67–10.63)	5.90 (3.15–10.00)	5.2 (3.02–8.33)	H = 5.19	0.08
Premature delivery *n* (%)^b^	6 (7.69)	27 (16.27)	12 (29.27)	*χ* ^2^=9.48	<0.01
Hospital stay (days) (IQR)^b^	10.00 (8.00–14.00)	11.00 (8.00–16.00)	15.00 (8.75–23.50)	H = 8.63	0.01
Clinic presentation *n* (%)					
Cough^a^	78 (100)	162 (97.69)	40 (97.59)	—	0.42
Wheezing^a^	60 (76.92)	123 (74.10)	31 (75.61)	*χ* ^2^=0.23	0.89
Dyspnea^c^ ^,^ ^b^	12 (15.38)	44 (26.51)	26 (63.41)	*χ* ^2^=31.25	<0.01
Cyanosis^d^ ^,^ ^b^	4 (5.13)	24 (14.46)	11 (26.83)	*χ* ^2^=10.92	<0.01
Lung moisture rales^a^	64 (82.05)	132 (79.52)	35 (85.37)	*χ* ^2^=0.80	0.67
Lung wheezing rales^a^	62 (79.49)	129 (77.71)	34 (82.93)	*χ* ^2^=0.56	0.76
Oxygen therapy^d^ ^,^ ^c^ ^,^ ^b^	12 (15.38)	46 (27.71)	26 (63.41)	*χ* ^2^=30.42	<0.01
PICU admission^d^ ^,^ ^c^ ^,^ ^b^	3 (3.85)	20 (12.05)	10 (24.39)	*χ* ^2^=11.16	<0.01
Mechanical ventilation^c^ ^,^ ^b^	1 (1.28)	9 (5.42)	9 (21.95)	*χ* ^2^=19.44	<0.01
Bronchoscopy observation *n* (%)					
Multiple malacia^d^ ^,^ ^c^ ^,^ ^b^	20 (25.64)	95 (57.23)	35 (85.37)	*χ* ^2^=42.23	<0.01
Sputum bolt^a^	2 (2.56)	15 (9.04)	2 (4.88)	—	0.18

^a^ No significant difference was observed in children with different extent of airway malacia.

^b^ Significant difference was observed in children with mild and severe airway malacia.

^c^ Significant difference was observed in children with moderate and severe airway malacia.

^d^ Significant difference was observed in children with mild and moderate airway malacia.

### 3.5 Pathogens

In children with airway malacia, the detection rate of at least one pathogen was higher than that in children without malacia (69.82% vs. 53.90%, *p* < 0.01). There were no significant differences in the pathogen detection rate among the children with different extents of airway malacia (*χ* ^2^ = 4.51, *p* = 0.10).

A total of 281 pathogens were detected in children with airway malacia: MP (30.18%) was the most common pathogen; RSV (15.79%) was the first viral pathogen detected, followed by hRV (10.88%), PIV III (7.02%), hBoV (5.26%), hMPV (2.46%), ADV (1.40%) and IV (1.40%). In children without airway malacia, MP (30.11%) was the most common pathogen, and the three most commonly detected viral pathogens were RSV (9.67%), hBoV (7.81%) and hRV (4.46%). The rates of RSV, hRV and PIV III positivity were higher in children with airway malacia than in those without airway malacia (all *p* < 0.05; Table 3).

**TABLE 3:** Pathogens identified from children with and without airway malacia.

Pathogen *n* (%)	With airway malacia (*n* = 285)	Without airway malacia (*n* = 269)	*χ* ^2^ test	*p*
MP	86 (30.18)	81 (30.11)	*χ* ^2^ =0.00	0.99
RSV	45 (15.79)	26 (9.67)	*χ* ^2^ =4.65	0.03
HRV	31 (10.88)	12 (4.46)	*χ* ^2^ =7.96	<0.01
hBoV	15 (5.26)	21 (7.81)	*χ* ^2^ =1.47	0.23
PIV III	20 (7.02)	7 (2.60)	*χ* ^2^ =5.82	0.02
hMPV	7 (2.46)	1 (0.37)	*χ* ^2^ =2.89	0.09
ADV	4 (1.40)	6 (2.23)	*χ* ^2^ =0.17	0.68
IV	4 (1.40)	1 (0.37)	*χ* ^2^ =0.70	0.40
HI	13 (4.56)	4 (1.49)	*χ* ^2^ =4.40	0.04
SP	19 (6.67)	17 (6.32)	*χ* ^2^ =0.03	0.87
MC	4 (1.40)	3 (1.12)	*χ* ^2^ =0.00	1.00
SA	7 (2.46)	1 (0.37)	*χ* ^2^ =4.23	0.04
Other bacteria	24 (8.42)	15 (5.58)	*χ* ^2^ =1.71	0.20

Abbreviations: ADV, adenovirus; HI, *Haemophilus influenzae*; hBoV, human bocavirus; hMPV, human metapneumovirus; HRV, human rhinovirus; IV, influenza virus; MC, *Moraxella catarrhalis*; MP, *Mycoplasma pneumoniae*; RSV, respiratory syncytial virus; SA, *Staphylococcus aureus*; SP, *Streptococcus pneumoniae*.

A total of 21.75% (62/285) of the children with airway malacia had positive BALF cultures, and the top three bacterial pathogens detected were *Streptococcus pneumoniae* (SP) (6.67%), *Haemophilus influenzae* (HI) (4.56%) and *Staphylococcus aureus* (SA) (2.46%). In children without airway malacia, the most common bacterial pathogens were SP, *Moraxella catarrhalis* (MC) and HI. The rates of HI and SA positivity were higher in children with airway malacia than in those without malacia (all *p* < 0.05; Table 3).

Mixed infections were identified in 24.91% (71/285) of the children with airway malacia and 17.47% (47/269) of the children without airway malacia, with statistically significant differences between the two groups (*χ* ^2^ = 4.57, *p* = 0.03). In the airway malacia group, 34 (47.89%), 25 (35.21%) and 13 (18.31%) patients had mixed viral‐MP, viral‐bacterial and bacterial‐MP infections, respectively. There was no significant difference in the detection rate of coinfections in children with different extents of malacia (*χ* ^2^ = 0.53, *p* = 0.77). In children without malacia, the top three coinfections were mixed viral‐MP, bacterial‐MP and viral‐bacterial coinfections.

## 4 DISCUSSION

In our study, we retrospectively analysed 554 children with persistent or recurrent pneumonia and found that 51.44% of the patients had airway malacia, which was consistent with previous research.^6^ In addition, our study revealed that 84.91% of children with airway malacia were under 1 year old, and the median age was 6 months. Additionally, Deacon et al.^12^ reported that the median age of children with airway malacia at diagnosis was 6.8 months. The results demonstrated that the possibility of airway malacia should be considered in children with persistent or recurrent pneumonia, especially those under 1 year of age.

Very few studies have reported on the correlation between airway malacia and the severity of recurrent or persistent pneumonia. In our study, children with airway malacia were more likely to receive oxygen therapy, mechanical ventilation and PICU admission than were those without malacia. These findings suggest that airway malacia likely aggravates the disease, which is consistent with the findings of Masters et al.^13^ suggesting that children with malacia have an increased possibility of severe respiratory illness and a tendency for delayed recovery. In children with airway malacia, both airway closure during cough and ineffective cough contribute to reduced clearance of mucus, which consequently worsens the clinical situation.^14^

We found that the incidences of dyspnoea, oxygen therapy, mechanical ventilation and PICU admission were higher in children with severe airway malacia than in those with mild or moderate malacia, which is consistent with the findings of Wei Pan et al.,^11^ indicating that severe malacia increases the severity of recurrent or persistent pneumonia compared to that in individuals with mild and moderate malacia. In addition, we found that there is an increased likelihood of multisite malacia in severe airway malacia patients. However, other studies have reported that the severity of malacia has a dose‐dependent effect on illness severity.^13^ In the future, more prospective studies with large sample sizes are needed to further explore the relationship between the extent of airway malacia and illness severity.

Currently, there is a paucity of research on the aetiology of pneumonia combined with airway malacia. In our study, MP was the most common pathogen, and RSV was the most common viral pathogen, which is consistent with the findings of previous studies.^15^, ^16^ In addition, the type of viral pathogen may be influenced by the season. In the present study, the rates of RSV, hRV, PIV III, HI, SA and mixed infection positivity were higher in children with airway malacia than in those without malacia, which may be related to the younger age of the children with airway malacia.

## 5 LIMITATIONS

This study had several limitations. First, this was a single‐centre retrospective study, and the incidence of airway malacia in children with recurrent or persistent pneumonia possibly increased. This may be related to the fact that the patients were limited to hospitalised children whose parents agreed to bronchoscopy. At the same time, as a retrospective study, some data, such as bronchiolitis in the first year of life, bronchial reactivity, delayed initiation of treatment or the implementation of correct therapy for bacterial pneumonia, were absent. Second, some of the viruses in this study were detected by direct immunofluorescence, which might have led to an underestimation of the actual positive rate due to the reduced sensitivity of the procedure. However, direct immunofluorescence offers a reliable alternative detection method with high specificity.

## 6 CONCLUSION

Airway malacia is one of the underlying causes of recurrent or persistent pneumonia. Airway malacia probably aggravates conditions. Compared with mild and moderate malacia, severe malacia worsens the severity of illness. There is an increased likelihood of multisite malacia in children with severe airway malacia. For younger children (especially those under 1 year old) with recurrent or persistent pneumonia, clinicians need to consider the possibility of airway malacia and perform bronchoscopy in a timely manner if conventional treatment is not effective.

## AUTHOR CONTRIBUTIONS

Yuqing Wang designed the research. Zhuxia Li, Xinxing Zhang and Huiquan Sun collected the data. Chuangli Hao conducted the statistical analysis. Xuejun Shao, Jun Xu and ZX were responsible virus detection and genomic analysis. Zhuxia Li wrote the initial manuscript and submitted the manuscript. All authors discussed the results and approved the final manuscript.

## CONFLICT OF INTEREST STATEMENT

The authors declare that they have no competing interests.

## ETHICS STATEMENT

The study was approved by the Ethics Committee of Childrens Hospital of Soochow University (No.2023CS038).

## PATIENT CONSENT STATEMENT

Consents of participation and publish were obtained from all patients or their families and this study was conducted in accordance with relevant guidelines and regulations.

## Data Availability

The datasets used and/or analysed during the current study are available from the corresponding author on reasonable request.

## References

[1] Wallihan R , Ramilo O . Community‐acquired pneumonia in children: current challenges and future directions. J Infect. 2014;69(Suppl 1):S87‐S90. doi:10.1016/j.jinf.2014.07.021 25264163

[2] Montella S , Corcione A , Santamaria F . Recurrent pneumonia in children: a reasoned diagnostic approach and a single centre experience. Int J Mol Sci. 2017;18(2):296. doi:10.3390/ijms18020296 28146079 PMC5343832

[3] Hoving MF , Brand PL . Causes of recurrent pneumonia in children in a general hospital. J Paediatr Child Health. 2013;49(3):E208‐E212. doi:10.1111/jpc.12114 23438187

[4] Chen LL , Liu YC , Lin HC , et al. Clinical characteristics of recurrent pneumonia in children with or without underlying diseases. J Formos Med Assoc. 2022;121(6):1073‐1080. doi:10.1016/j.jfma.2021.08.013 34454794

[5] Heffelfinger JD , Davis TE , Gebrian B , Bordeau R , Schwartz B , Dowell SF . Evaluation of children with recurrent pneumonia diagnosed by World Health Organization criteria. Pediatr Infect Dis J. 2002;21(2):108‐112. doi:10.1097/00006454-200202000-00005 11840076

[6] Santiago‐Burruchaga M , Zalacain‐Jorge R , Vazquez‐Cordero C . Are airways structural abnormalities more frequent in children with recurrent lower respiratory tract infections. Respir Med. 2014;108(5):800‐805. doi:10.1016/j.rmed.2014.02.014 24709380

[7] Gokdemir Y , Cakir E , Kut A , et al. Bronchoscopic evaluation of unexplained recurrent and persistent pneumonia in children. J Paediatr Child Health. 2013;49(3):E204‐E207. doi:10.1111/jpc.12124 23438344

[8] Williamson A , Young D , Clement WA . Paediatric tracheobronchomalacia: incidence, patient characteristics, and predictors of surgical intervention. J Pediatr Surg. 2022;57(11):543‐549. doi:10.1016/j.jpedsurg.2022.05.005 35718546

[9] Petreschi F , Coretti A , Porcaro F , et al. Pediatric airway compression in aortic arch malformations: a multidisciplinary approach. Front Pediatr. 2023;11:1227819. doi:10.3389/fped.2023.1227819 37547103 PMC10401269

[10] Koumbourlis AC , Belessis Y , Cataletto M , et al. Care recommendations for the respiratory complications of esophageal atresia‐tracheoesophageal fistula. Pediatr Pulmonol. 2020;55(10):2713‐2729. doi:10.1002/ppul.24982 32716120

[11] Pan W , Peng D , Luo J , et al. Clinical features of airway malacia in children: a retrospective analysis of 459 patients. Int J Clin Exp Med. 2014;7(9):3005‐3012. 25356175 PMC4211825

[12] Deacon J , Widger J , Soma MA . Paediatric tracheomalacia—a review of clinical features and comparison of diagnostic imaging techniques. Int J Pediatr Otorhinolaryngol. 2017;98:75‐81. doi:10.1016/j.ijporl.2017.04.027 28583509

[13] Masters IB , Zimmerman PV , Pandeya N , Petsky HL , Wilson SB , Chang AB . Quantified tracheobronchomalacia disorders and their clinical profiles in children. Chest. 2008;133(2):461‐467. doi:10.1378/chest.07-2283 17989148

[14] Wallis C , Alexopoulou E , Antón‐Pacheco JL , et al. ERS statement on tracheomalacia and bronchomalacia in children. Eur Respir J. 2019;54(3):1900382. doi:10.1183/13993003.00382-2019 31320455

[15] Vervloet LA , Marguet C , Camargos PA . Infection by mycoplasma pneumoniae and its importance as an etiological agent in childhood community‐acquired pneumonias. Braz J Infect Dis. 2007;11(5):507‐514. doi:10.1590/s1413-86702007000500012 17962878

[16] Yu‐Qing W , Chuang‐Li H , Wei J , Zheng‐Rong C , Xin‐Xin Z , Wen‐Jing G . Etiology and clinical characteristics of community‐acquired pneumonia with airway malacia in children. J Trop Pediatr. 2018;64(4):317‐325. doi:10.1093/tropej/fmx071 29036724

